# Genetic and Epigenetic Modulation of Growth Hormone Sensitivity Studied With the IGF-1 Generation Test

**DOI:** 10.1210/jc.2015-1413

**Published:** 2015-04-02

**Authors:** Meriem Ouni, Anne-Laure Castell, Agnès Linglart, Pierre Bougnères

**Affiliations:** Institut National de la Santé et de la Recherche Médicale Unité 986 (M.O., A.-L.C., A.L., P.B.) and Department of Pediatric Endocrinology and Diabetes (A.-L.C., A.L., P.B.), Paris Sud University, Bicêtre Hospital, 94275 Le Kremlin-Bicêtre, France

## Abstract

**Context:**

Like all hormones, GH has variable physiological effects across people. Many of these effects initiated by the binding of GH to its receptor (GHR) in target tissues are mediated by the expression of the *IGF1* gene. Genetic as well as epigenetic variation is known to contribute to the individual diversity of GH-dependent phenotypes through two mechanisms. The first one is the genetic polymorphism of the *GHR* gene due to the common deletion of exon 3. The second, more recently reported, is the epigenetic variation in the methylation of a cluster of CGs dinucleotides located within the proximal part of the P2 promoter of the IGF-1 (*IGF1*) gene, notably CG-137.

**Objective:**

The current study evaluates the relative contribution of these two factors controlling individual GH sensitivity by measuring the response of serum IGF-1 to a GH injection (IGF-1 generation test) in a sample of 72 children with idiopathic short stature.

**Results:**

Although the d3 polymorphism of the GHR contributed 19% to the variance of the IGF-1 response, CG-137 methylation in the *IGF-1* promoter contributed 30%, the combined contribution of the two factors totaling 43%.

**Conclusion:**

Our observation indicates that genetic and epigenetic variation at the *GHR* and *IGF-1* loci play a major role as independent modulators of individual GH sensitivity.

Tens of thousands of children affected by various causes of short stature currently receive recombinant GH to improve their final height. There is, however, a highly variable individual GH response to this treatment, which does not allow pediatric endocrinologists to predict final height reliably, although clinical predictive models have been developed (1) as well as biological models based on GH/IGF-1 hormones measured before treatment (2). The multiple causes underlying the variability of the therapeutic response to GH include the etiology of short stature (3, 4), GH dose (5), child age (1), treatment regimens (6), and therapeutic compliance (7). Because IGF-1 is the mediator of GH effects, the individual variation in growth responses is in large part due to the variable IGF-1 production under GH treatment, reflected by circulating IGF-1 concentration (8–10). On the other hand, the personal capacity to respond to GH has prompted few studies in search of biological mechanisms able to vary across individuals (11, 12). The increment in IGF-1 induced by GH administration in healthy children with idiopathic short stature is normally distributed (8, 10, 13, 14) and can thus be modeled as a continuous quantitative trait. Among the molecular determinants involved in the variability of IGF-1 responses to GH, it has become clear that genetic variation plays a role (11). Notably, the deletion of exon 3 within the GH receptor (*GHR*d3) gene has been recognized as a significant predictor of GH growth-promoting effects in children with idiopathic short stature (15–18) or other etiologies of short stature (11, 12, 19).

Estimates of the proportion of variance in circulating IGF-1 that is genetically determined vary between 38% and 80% (20, 21). The association of serum IGF-1 concentration with several genetic variants is debated (22–24), but variants at the *IGF-1* locus do not seem to influence circulating IGF-1 in Caucasian adults (22, 25) except, perhaps, the most common Z allele of the microsatellite located 1 kb upstream the *IGF-1* gene (26, 27). Overall, the genetic basis for serum IGF-1 variability remains largely unknown in adults and has not been studied during the period of physiological growth in normal children. In children with idiopathic short stature, considered to be a variant category of normal children, the *GHR*d3 is associated with higher circulating IGF-1 in response to GH injection (28) .

Unlike pharmacogenetics, pharmacoepigenetics is a nascent field of clinical medicine (29), and the epigenetics of growth and IGF-1 responses have only recently started to be investigated (30). A recent study showed that the methylation of a cluster of dinucleotides (CGs) located within the P2 promoter of the IGF-1 (*IGF-1*) gene, notably CG-137, is inversely closely correlated with the response of growth and circulating IGF-1 to GH treatment (30).

In growing children, GH responsiveness is important to physiology and in some of them to therapeutics. The current study investigates the individual response of children to a GH injection with the objective of evaluating the respective role of genetic polymorphism of the *GHR* and the degree of methylation of the P2 promoter of the *IGF-1* gene. We selected children who have not entered puberty to avoid the confounding effect of the variable tempo of sexual maturation, which adds to the variability of basal (31–33) and GH-stimulated (34) circulating IGF-1. We used the long-studied generation test (31, 34–37) to study the GH responsiveness of circulating IGF-1 in 72 children. We studied the GH receptor (GHR) d3 genotype and the methylation of the IGF-1 P2 promoter in these children to study how these factors contribute to the individual variability of the GH response to the test.

## Materials and Methods

### Participants

Seventy-two children with various degrees of short stature (−1.1 SD to −3.2 SD) belonging to the Epigrowth cohort (30) had venous blood sampling at 8:00 pm before dinner, 10 minutes before receiving an injection of 100 μg/kg body weight of recombinant human growth hormone (rhGH) im in the left thigh, had a normocaloric standard dinner at 8:10 pm, fasted overnight, and had a second blood sampling at 8:00 am. Their main characteristics are depicted in Table 1. All children were healthy and had normal clinical examination. Thirty children had criteria of idiopathic short stature, as defined in (11). In these children, GH deficiency was excluded with a stimulated GH peak greater than 15 ng/mL, TSH levels were normal. Subtle chondrodysplasia were excluded by forearm, pelvis, and spine radiographs. Pubertal stages were estimated using the Tanner definition.

**Table 1.** Main characteristics of the studied children, mean ± SD

	Acute rhGH Injection test
n	72
Sex, M/F	45/27
Age, y	11 ± 2.5
Height (SDS)	−1.8 ± 0.7
Tanner stages, n	
1	41 (57%)
2	20 (28%)
3	10 (14%)
4	1 (1%)
Serum IGF-1 before test	
Nanograms per milliliter	236 ± 149
SDS	−1.16 ± 0.8
Serum IGF-1 12 h after rhGH	
Nanograms per milliliter	326 ± 150
SDS	−0.55 ± 0.84
*GHR* genotype	
fl/fl	33 (46%)
fl/d3	32 (44%)
d3/d3	7 (10%)
*IGF-1* P2 promoter methylation, %	
CG-232	62 ± 6
CG-224	74 ± 7
CG-218	72 ± 6
CG-207	45 ± 7
CG-137	47 ± 4
CG-108	60 ± 6
Average^a^	58 ± 4

Abbreviations: F, female; M, male.

a Mean value for the six studied CGs.

Parents of all studied children gave their written informed consent for the study according to the French rules of bioethics in biomedical research checked by our institutional review board.

### Serum IGF-1 and IGF binding protein-3 (IGFBP3) concentrations

Serum IGF-1 concentration was measured at approximately 7:00–8:00 am before breakfast in 136 children using an immune-radiometric assay after ethanol-acid extraction using Cisbio reagents. Intra- and interseries coefficients of variation were 1.5% and 3.7% at 260 ng/mL and 3.9% at 760 ng/mL. The sensitivity was 4 ng/mL. IGF-1 SD score (SDS) was calculated using the norms of Alberti et al (33) in French children. Serum IGFBP3 was measured using an immune-radiometric assay after ethanol-acid extraction with Diagnostic Systems Laboratory reagents (Beckman-Coulter). Intra- and interseries coefficient of variation was 10.4 and 14% and the sensitivity limit was 6.2 ng/mL.

### *GHR* genotype

Analysis of the *GHR* exon 3 polymorphism was carried out in patients by quantitative PCR performed on ABI 7500 fast (Applied Biosystems). Genotyping protocol was adapted from Bernabeu et al (38). Primer/probe sets were targeted to exon 3 and exon 10 of *GHR*, which was used as an internal positive control. We used oligonucleotide primers and probes previously described in (38).

For each analysis, 50 ng genomic DNA was quantitative PCR amplified in 96-well plates in a volume of 12 μL using Gotaq probe quantitative PCR master mix (Promega), 1.25 μL *GHR* exon 3 primer/probe set (3 pm/μL each), 1.25 μL *GHR* exon 10 primer/probe set (9 pm/μL each), and 2.75 μL sterile water. Cycle conditions were 50ºC for 2 minutes and 95ºC for 10 minutes, followed by 40 cycles of 95ºC for 15 seconds and 60ºC for 1 minute. Differences in cycle threshold (Ct) between the exon 3 and exon 10 amplicons were used to determine the exon 3 copy number for each sample. A δCt value of 1 indicates two exon 3 copies (genotype fl/fl), a δCt value of 2 indicates one exon 3 copy (genotype fl/d3), and no signal for exon 3 in the presence of a normal exon 10 signal indicates an absent exon 3 (genotype d3/d3).

### DNA methylation

For methylation measurements, 6 mL peripheral blood samples were obtained, from which peripheral blood mononuclear cells were purified immediately.

For promoter P2, we studied the six CGs located upstream from the major transcription start site within the proximal part of the P2 promoter. CGs are denominated according to position vs each promoter transcription start site. Nucleic acids were extracted from peripheral blood mononuclear cells using Gentra Puregene blood kit (QIAGEN). A bisulfite-PCR-pyrosequencing technique (39) was used to measure the methylation of the CGs. We improved the resolution of this method from a handful of bases to up to 100 nucleotides, with the ability to quantify methylation in the same sample of blood with a coefficient of variation (SD/mean) as little as 1%–5%. Briefly, 400 ng of genomic DNA was treated with an EZ DNA Methylation-Gold kit, according to manufacturer's protocol (Zymo Research Corp). The bisulfite-treated genomic DNA was PCR amplified using unbiased *IGF-1* primers (30) and performed quantitative pyrosequencing using a PyroMark Q96 ID Pyrosequencing instrument (QIAGEN). Pyrosequencing assays were designed using MethPrimer (http://www.urogene.org/methprimer/index1.html). Biotin-labeled single-stranded amplicons were isolated according to protocol using the QIAGEN Pyromark Q96 Work Station and underwent pyrosequencing with 0.5 μM primer. The percentage methylation for each of the CGs within the target sequence was calculated using PyroQ CpG Software (QIAGEN).

### Calculations and statistics

The IGF-1 response to GH administration was expressed as increment in serum IGF-1 concentration, ie, the difference between serum IGF-1 12 hours after injecting GH and serum IGF-1 concentration before GH injection. Pearson correlations were calculated as adjusted R square that measures the proportion of the variation in the dependent variable accounted for by the explanatory variables. The fraction of explained variance is calculated under the linear regression model, using the usual definition: r^2^ × 100. We fitted a multivariate linear model to the data to estimate the proper effect of *GHR* genotype and CG-137 methylation on response to GH, adjusted for the effect of the other covariates contributing to the IGF-1 response, such as age and sex. We carried out tests of independence of each covariate one at a time, keeping the others in the model. Statistics and estimations of effect given in the tables are thus adjusted for the others whenever appropriate and are not subject to marginal association. We checked the normality of the residuals, and the residuals vs the fitted values did not show any trend, indicating that there was no noticeable deviation from the assumption of the linear model. All statistics and linear model were computed using R 2.10.1. Results are expressed as mean ± SD.

## Results

The response of serum IGF-1 concentration to the GH test averaged 90 ± 54 ng/mL, an increase of 38 ± 36% from the basal IGF-1 value. There was no correlation between basal IGF-1 and the increase of IGF-1 during the test. Within the studied group of children, the IGF-1 response to GH showed a nonsignificant trend of positive association with age (*P* = .11) and Tanner pubertal stages (*P* = .45).

**Figure 1.**
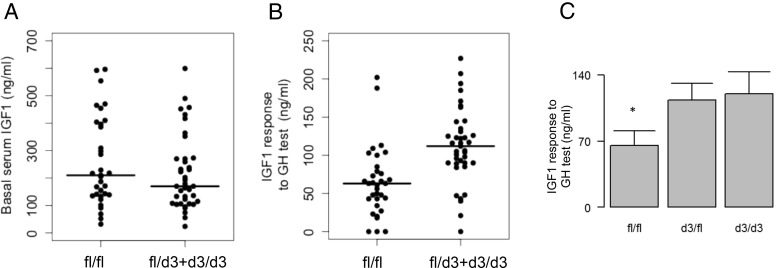
Relationship between GHR-d3 genotype and serum IGF-1 concentration in the studied children. A, Basal IGF-1 shows no difference across the genotypic groups. B, The response of serum IGF-1 to GH test is greater in children carrying a d3 allele (*P* = 2.10^−5^). C, Mean values of IGF-1 response in the three GHR genotypic groups show that d3/fl and d3/d3 classes have a comparable response to GH.

Basal IGF-1 concentration was comparable in children of various GHR genotypes (Figure 1A), but a significant difference appeared after the GH injection (Figure 1B). Children carrying the dominant *GHR*-allele d3 (15) had a 56% higher IGF-1 response to GH than the fl/fl homozygotes. Because d3/3 children and fl/d3 showed comparable IGF-1 responses to GH, they were merged in a single group for analysis.

**Figure 2.**
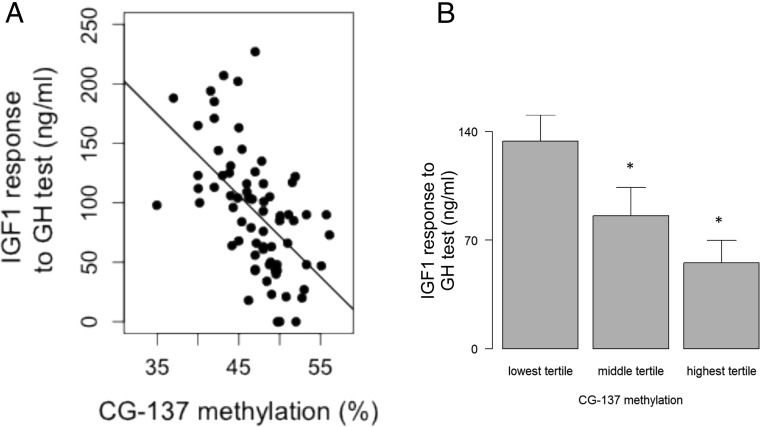
Relationship between CG-137 methylation and IGF-1 response to the GH test. A, Correlation between serum IGF-1 and CG-137 methylation (R = −0.54, *P* = 4.10^−7^, y = −6.8X + 413). B, IGF-1 response across the three tertiles of CG-137 methylation (*P* = 10^−6^).

The serum IGF-1 response to the GH test showed a negative correlation with the methylation of three of six CGs of the IGF-1 P2 promoter (Supplemental Table 1), notably with CG-137 methylation (R = −0.54, *P* = 4.10^−7)^ as shown in Figure 2A. Children in the highest tertile for CG-137 methylation showed a 142% increase in IGF-1 response compared with those in the lowest tertile (Figure 2B). CG methylation was comparable in the GHR genotype groups.

**Figure 3.**
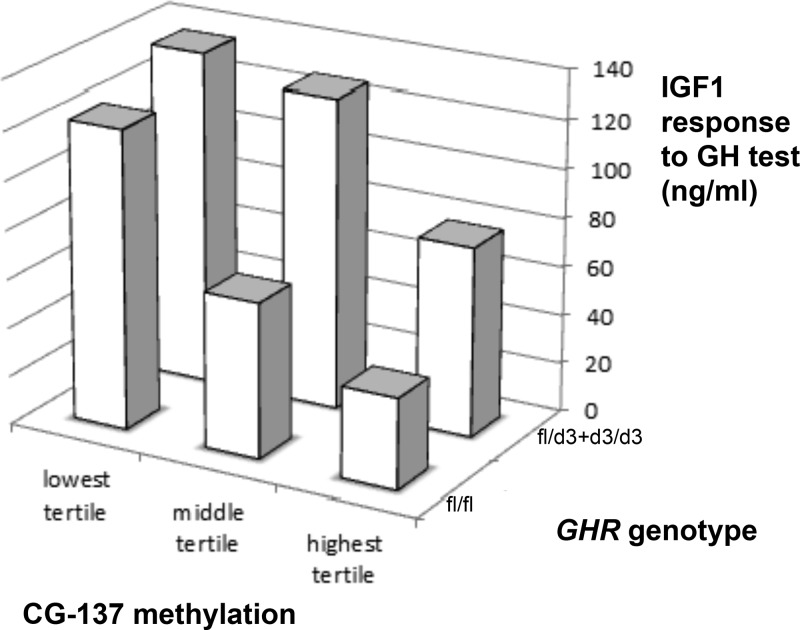
3-D relationship between IGF-1 response to GH test and both GHR-d3 genotype and CG-137 methylation showing higher responses in children carrying a d3 allele and having low levels of CG-137 methylation (*P* = 10^−9^).

Once combined in a multivariate analysis with age, pubertal stages, and sex, both GHR d3 genotype and the methylation of CG-137 showed a significant association with the serum IGF-1 response to the GH test (Table 2). The combined effect of GHR genotype and IGF-1 epigenotype is illustrated by the three-dimensional histograms of Figure 3. Overall, the GHR d3 genotype contributed 19% to the variance of the IGF-1 response and the *IGF-1* P2 methylation contributed 30%, resulting in a total contribution of 43% to the individual GH sensitivity evaluated by the IGF-1 generation test. Strikingly, the children who had both a GHR fl/fl genotype and a CG-137 methylation in the highest tertile (n = 18) had an IGF-1 response to the GH test 3.4 times smaller than children carrying one or two GHR d3 alleles combined with CG-137 methylation in the lowest tertile (n = 11) (Figure 3).

**Table 2.** General linear model for regression of age, sex, Tanner stage, *GHR* d3 polymorphism, and methylation of CG-137 on IGF-1 response to the GH test

	Estimates	SE	t Value	Pr (> t )
Intercept	275	58	4.7	10^−5^
Age, y	6.3	2.74	2.30	0.025
Sex	15.03	10.3	1.46	0.15
Tanner stage (1–4)	−12.84	9.3	−1.40	0.17
*GHR* d3 polymorphism	35.7	9.20	3.87	2.10^−4^
CG-137 methylation, %	−5.60	1.13	−4.95	5.6.10^−6^

Pr (> t), probability of a value > t.

We found no correlation of serum IGFBP3 with the GHR genotype or with the IGF-1 P2 promoter methylation, in the basal state as well as in response to the GH test.

## Discussion

Our study supports that both the GHR genotype and the CG methylation of the P2 promoter of the *IGF-1* gene are major determinants of the individual IGF-1 response to GH in childhood.

IGF-1 generation tests were developed more than 20 years ago (31, 34–37) and are currently used in evaluating GH sensitivity in children with unexplained short stature, notably when characterized by low serum IGF-I. Major limitations have included variability in protocols for administration of GH, timing of samples, differences in IGF assay methodologies, and lack of adequate normative data (40). The latter is particularly problematic, given the well documented age-related variability in IGF-I concentrations (12, 32) as well as gender-related differences in responsiveness to exogenous GH (41, 42). The results of the generation test in children with idiopathic short stature are of particular interest, as previously reported (36), to have serum IGF-I concentrations in the lower portion of the normal range or below the lower limits of normal. Interestingly, these subjects also failed, in general, to raise their serum IGF-1 concentrations in response to GH; many did not even attain levels within the baseline normal range. According to another report (34), this was previously considered to result from mild mutations of the *GHR* gene or from subtle postreceptor mechanisms (36, 43, 44). The current results establish the GHR d3 genotype and the *IGF-1* P2 epigenotype as major sources of individual variation of the IGF-1 generation test. This can be of importance for understanding the phenotypic diversity in GH sensitivity at the individual level.

The vast majority of the increase in serum IGF-1 concentration occurring in response to GH is considered to result from GH effects on *IGF-1* expression in child liver, mediated by GH binding to its hepatic receptor. A weakness of our study is that we could not study the association of liver P2 methylation with GH responsiveness. Such lack of availability and analysis of epigenomes in specific cells of physiological tissues is a common but major limitation of epigenetic epidemiology (45, 46), which most often has to rely on blood cells (47, 48) .

The expression of the *IGF-1* gene in liver is controlled by GH at a transcriptional level. In mammals including humans, the *IGF-1* gene is composed of six exons and five introns that span greater than 80 kb of chromosomal DNA (49, 50). Tandem promoters direct *IGF-1* gene transcription through unique leader exons. Promoter 1, which uses heterogeneous transcription initiation sites, is active in multiple animal tissues (51), whereas the smaller and simpler promoter 2 is primarily but not exclusively active in the liver of cattle (52), unlike in rodents in which promoter 2 activity seems exclusively hepatic (14). GH exerts its effects through the Janus kinase/signal transducer and activator of transcription pathway with translocation of activated signal transducer and activator of transcription-5b transcription factor to the nucleus in which it regulates *IGF-1* transcription (53). In the liver of hypophysectomized rats, GH induces dramatic changes in chromatin at the *IGF-1* locus and activates *IGF-1* transcription by distinct promoter-specific epigenetic mechanisms (54, 55). Yet not only CG location and composition are different in human and rat *IGF-1* promoters, but also the pattern of methylation and its transcriptional effects on rat promoters are still unknown.

Although the d3 polymorphism at the GHR locus and the variation of methylation at the IGF-1 P2 promoter account for a total 43% of the individual variability of GH effects on serum IGF-1, the remaining 57% of variance are yet to be explained by other factors. Age, puberty, or sex could be important but do not play a significant role in the current group of children recruited within a relatively narrow age range. Because serum IGF-1 concentration mostly reflects IGF-1 production by the liver in response to GH, it is possible that some of the variance comes from the factors controlling GH pharmacokinetics or from the molecular pathway involved in the hepatic signaling of GH upstream IGF-1 production. This pathway encompasses the *GHR, JAK2*, and *STAT5b* gene products whose expression can vary, depending on individual genotypes and epigenotypes. We are not aware that genome-wide association studies or methylome-wide association studies for serum IGF-1 in children (basal or in response to GH) have yet been carried out. The challenge of understanding the remaining part of individual GH sensitivity thus remains entire.

Our observation indicates that genetic and epigenetic variation at the *GHR* and *IGF-1* loci play a major role as independent modulators of individual GH sensitivity.
